# From Waste to Biocatalyst: Cocoa Bean Shells as Immobilization Support and Substrate Source in Lipase-Catalyzed Hydrolysis

**DOI:** 10.3390/molecules30153207

**Published:** 2025-07-30

**Authors:** Luciana Lordelo Nascimento, Bruna Louise de Moura Pita, César de Almeida Rodrigues, Paulo Natan Alves dos Santos, Yslaine Andrade de Almeida, Larissa da Silveira Ferreira, Maira Lima de Oliveira, Lorena Santos de Almeida, Cleide Maria Faria Soares, Fabio de Souza Dias, Alini Tinoco Fricks

**Affiliations:** 1 Programa de Pós-Graduação em Ciência de Alimentos, Universidade Federal da Bahia, Salvador 40170-115, BA, Brazil; lucianalordelo@outlook.com (L.L.N.); lsferreira03@gmail.com (L.d.S.F.); almeidalorena@ufba.br (L.S.d.A.); 2 Departamento de Análises Bromatológicas, Faculdade de Farmácia, Universidade Federal da Bahia, Salvador 40170-115, BA, Brazil; brunalmpita@gmail.com (B.L.d.M.P.); mairalimaoli@hotmail.com (M.L.d.O.); 3 Programa de Pós-Graduação em Biotecnologia Industrial, Universidade Tiradentes, Aracaju 49032-490, SE, Brazil; cesaralmeidar@gmail.com (C.d.A.R.); cleide18@yahoo.com.br (C.M.F.S.); 4 Rede Nordeste de Biotecnologia, Universidade Federal de Sergipe, São Cristóvão 49107-230, SE, Brazil; natan-9@hotmail.com; 5 Programa de Pós-Graduação em Química, Universidade Federal de Sergipe, São Cristóvão 49107-230, SE, Brazil; yslaineandrade@live.com; 6 Programa de Pós-Graduação em Química, Universidade Federal da Bahia, Salvador 40170-115, BA, Brazil; fsdias@ufba.br

**Keywords:** cocoa bean shells, immobilization, lipase, cocoa bean shell oil, free fatty acids

## Abstract

This study reports the development of a sustainable biocatalyst system for free fatty acid (FFA) production from cocoa bean shell (CBS) oil using *Burkholderia cepacia* lipase (*BCL*). CBS was explored as both a support material and a reaction substrate. Six immobilized systems were prepared using organic (CBS), inorganic (silica), and hybrid (CBS–silica) supports via physical adsorption or covalent binding. Among them, the covalently immobilized enzyme on CBS (ORG-CB) showed the most balanced performance, achieving a catalytic efficiency (Ke) of 0.063 mM^−1^·min^−1^ (18.6% of the free enzyme), broad pH–temperature tolerance, and over 50% activity retention after eight reuse cycles. Thermodynamic analysis confirmed enhanced thermal resistance for ORG-CB (Ed = 32.3 kJ mol^−1^; ΔH‡ = 29.7 kJ mol^−1^), while kinetic evaluation revealed that its thermal deactivation occurred faster than for the free enzyme under prolonged heating. In application trials, ORG-CB reached 60.1% FFA conversion from CBS oil, outperforming the free enzyme (49.9%). These findings validate CBS as a dual-function material for enzyme immobilization and valorization of agro-industrial waste. The results also reinforce the impact of immobilization chemistry and support composition on the operational and thermal performance of biocatalysts, contributing to the advancement of green chemistry strategies in enzyme-based processing.

## 1. Introduction

Triacylglycerols (TAGs), which serve as the primary form of energy storage, are widely present in the seeds of many eukaryotic plants. During seed germination, TAGs are hydrolyzed by lipases into free fatty acids (FFAs) and glycerol derivatives. These FFAs are subsequently metabolized via oxidative catabolism, providing both energy and molecular precursors necessary for seedling growth. Lipases, as key triglyceride hydrolases, are primarily responsible for TAG hydrolysis but also exhibit broad catalytic versatility, enabling reactions such as esterification, transesterification, alcoholysis, acidolysis, aminolysis, and deacetylation [1,2,3].

The lipase from *Burkholderia cepacia* (*BCL*), an extracellular enzyme produced by this Gram-negative bacterium, is a versatile biocatalyst widely used in biotechnological applications—ranging from fatty acids production and detergent formulations to the kinetic resolution of racemic compounds—due to its broad substrate specificity and stability under diverse conditions [2,3]. Despite these advantages, the use of free lipases in industrial processes is often hampered by limited operational stability, difficulty in recovery, and rapid activity loss during prolonged use [1].

To overcome these drawbacks, enzyme immobilization on solid supports has emerged as a widely adopted strategy [1]. By confining enzymes onto carriers through physical or chemical interactions, immobilization enhances resistance to heat, pH fluctuations, and denaturants, while also facilitating enzyme recovery and reuse. This approach is essential to improving the economic feasibility and robustness of biocatalytic systems. The performance of immobilized enzymes is known to depend strongly on both the immobilization method and the physicochemical properties of the support material [1,2,4].

Various support types have been investigated, including inorganic matrices (e.g., silica and oxides) and synthetic polymers, which offer high mechanical strength and reproducibility. However, recent efforts have shifted toward renewable, low-cost, and eco-friendly supports that enhance both enzyme activity and sustainability [1,4,5]. In this context, lignocellulosic agro-industrial residues have gained increasing attention as promising carriers for enzyme immobilization [5].

These materials are abundant, inexpensive, and easily functionalized, with favorable characteristics such as high surface area, mechanical robustness, chemical inertness, and process stability [1,5,6]. Moreover, using agricultural waste as a support not only reduces production costs but also contributes to sustainability by valorizing otherwise discarded biomass. Several studies have successfully demonstrated the use of lipases immobilized on lignocellulosic materials—including coconut fiber, guava seed biochar, rice husk ash, and spent coffee grounds—for fatty acids production or biodiesel production, achieving favorable performance and cost-effectiveness [1].

Cocoa bean shells (CBSs) represent a particularly attractive candidate in this context. These are the fibrous husks removed from cocoa beans during chocolate processing, accounting for approximately 10% of the bean’s weight [7]. As chocolate production depends on large-scale cocoa bean processing, this byproduct is generated in significant amounts. In 2022, approximately 5020 thousand metric tons of cocoa were ground worldwide, posing both environmental and economic challenges for CBS disposal. Rich in lignocellulose (~50% fiber) and containing proteins, minerals, polyphenols, methylxanthines, and residual cocoa butter (~11% of dry weight), CBS offers potential as both support material and reaction substrate [7,8,9].

Despite its promising characteristics, the use of cocoa bean shells (CBSs) in enzyme immobilization remains poorly explored. To the best of our knowledge, no previous studies have investigated CBSs as a dual-function material—where the solid biomass serves as a support for enzyme immobilization, while the oil extracted from CBS acts as the substrate for enzymatic hydrolysis. This integrated approach offers a novel and sustainable biocatalytic platform that aligns with circular economy principles by fully valorizing agro-industrial waste.

In this context, the present study investigates the immobilization of *Burkholderia cepacia* lipase using CBS as an organic support. Its performance is compared with two additional systems: an inorganic support (silica) and a hybrid composite composed of CBS and silica. Each material is tested with two immobilization techniques—physical adsorption and covalent binding—to evaluate the influence of support composition and immobilization strategy on the resulting biocatalyst’s performance. The most promising system is further applied in the enzymatic hydrolysis of CBS oil, demonstrating its catalytic potential in converting waste-derived lipids into free fatty acids.

## 2. Results and Discussion

### 2.1. Evaluation and Selection of the Optimal Immobilized Lipase System

This section presents the performance of *BCL* in its free form and immobilized on three different supports—organic (ORG), hybrid (HYB), and inorganic (INO)—using either physical adsorption (PA) or covalent binding (CB). For clarity, the immobilized systems are designated as follows: ORG-PA and ORG-CB for physical adsorption and covalent binding on the organic support, respectively; HYB-PA and HYB-CB for the hybrid support; and INO-PA and INO-CB for the inorganic support.

The results are discussed in terms of immobilization yield, pH and temperature activity profiles, kinetic parameters, and operational stability, aiming to identify the most effective biocatalyst for fatty acid production from cocoa bean shell oil.

#### 2.1.1. Immobilization Yield

**Figure 1:**
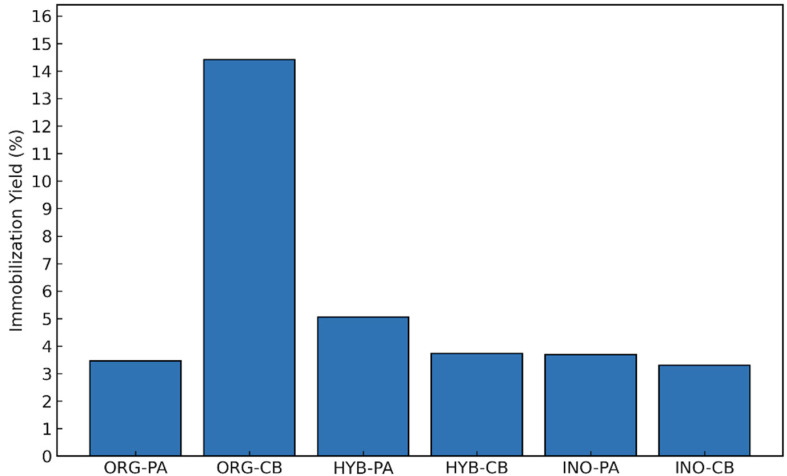
Immobilization yield of *Burkholderia cepacia* lipase (*BCL*) on organic (ORG), hybrid (HYB), and inorganic (INO) supports via physical adsorption (PA) and covalent binding (CB).

The immobilization yield, expressed as the percentage of enzymatic activity recovered after attachment to the support, offers important insight into the effectiveness of the immobilization process. It reflects both the amount of enzyme successfully bound and the preservation of its catalytic activity post-immobilization. In this study, yields ranged from 3.3 to 14.4%, depending on the immobilization method and support type (Figure 1).

Among all systems, ORG-CB (covalently immobilized lipase on the organic support) exhibited the highest recovery yield at 14.1%, indicating that covalent binding to the lignocellulosic matrix enabled the most efficient enzyme attachment. In contrast, the physically adsorbed counterpart ORG-PA achieved a recovery yield of only 3.5%, suggesting weaker interactions with the support and possible enzyme leaching during the immobilization process. The other systems presented low recovery rates overall, likely due to the lower biocompatibility and the reduced number of functional groups available for stable enzyme binding. Additionally, the superior performance of the organic support aligns with its lignocellulosic structure, which offers abundant functional groups and a favorable surface for enzyme interaction [5].

These findings confirm that the immobilization method and the chemical nature of the support play critical roles in determining enzyme retention. This behavior can be attributed to the distinct immobilization mechanisms involved. In covalent systems, the CBS support was first functionalized with APTES and activated with glutaraldehyde. Immobilization likely occurred via Schiff base formation between aldehyde groups (–CHO) introduced by glutaraldehyde and primary amine groups (–NH_2_) on surface-exposed lysine residues of the enzyme. These imine linkages contribute to strong, stable enzyme attachment and reduced desorption.

In contrast, in physical adsorption systems (PA), no chemical modification was applied to the CBS support. In these systems, immobilization relies on non-covalent interactions such as hydrogen bonding (e.g., between hydroxyl groups on cellulose or hemicellulose and polar amino acid residues), hydrophobic interactions (involving lignin domains and hydrophobic patches on the enzyme surface), and electrostatic interactions depending on the pH and surface charge distribution. These weaker interactions are more susceptible to enzyme leaching, especially under operational conditions.

Covalent immobilization has often been reported to outperform physical adsorption in terms of yield. For instance, Melo et al. [10] observed significantly higher protein retention and catalytic activity in covalently immobilized systems compared to those relying on weaker interactions such as adsorption or entrapment.

Almeida et al. [4] reported the covalent immobilization of lipase on silica, achieving an immobilization yield of 12%, which is comparable to the best-performing system in the present study (ORG-CB). To improve this yield, the authors incorporated polyethylene glycol (PEG) into the immobilization protocol, which significantly increased the yield to 89%, highlighting PEG as an effective strategy for enhancing enzyme retention. Similarly, Panjaitan et al. [11] improved lipase immobilization by using nickel foam as a support, benefiting from the strong covalent interactions formed between nickel ions and the enzyme. Therefore, although the immobilization yields achieved in this study were modest, they could potentially be enhanced by adopting additional strategies such as support modification, use of activation agents (e.g., PEG), or metal ion coordination.

While the immobilization yield is a critical starting point, subsequent sections demonstrate that catalytic activity, kinetic behavior, and reusability must also be considered for selecting the optimal biocatalyst system.

#### 2.1.2. Effect of pH on Hydrolytic Activity

The pH of the environment significantly influences enzyme activity by altering the ionization states of amino acid residues within the enzyme’s active site. These changes can modify the enzyme’s three-dimensional structure, affecting substrate binding and catalytic efficiency. For instance, variations in pH can shift the balance of protonated and deprotonated forms of active site residues, leading to conformational changes that impact enzymatic function. Understanding these effects is crucial for optimizing enzymatic reactions in various biochemical applications [12].

**Figure 2:**
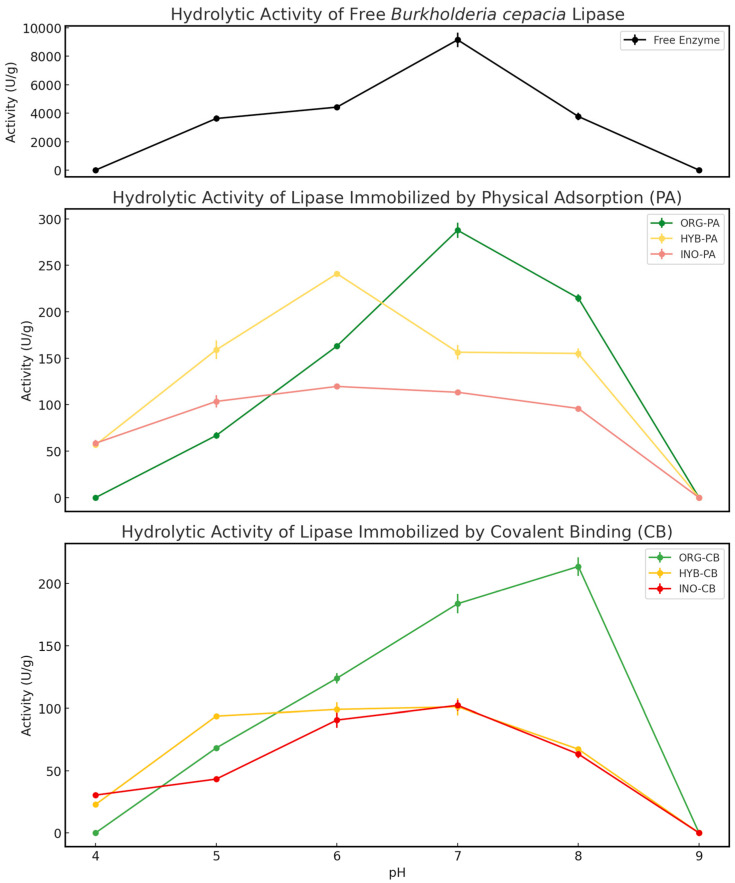
Effect of pH on the activity of free and immobilized *Burkholderia cepacia* lipase (*BCL*) on organic (ORG), hybrid (HYB), and inorganic (INO) supports via physical adsorption (PA) and covalent binding (CB).

The hydrolytic activity of *BCL* in its free and immobilized forms was evaluated across a pH range from 4.0 to 9.0 (Figure 2). The free enzyme exhibited a sharp activity peak at pH 7.0, reaching its maximum value of 9145.6 U g^−1^, which was defined as 100% relative activity. Activity declined significantly under both acidic and mildly alkaline conditions, dropping to 48% (4422 U g^−1^) at pH 6.0, 41% (3766 U g^−1^) at pH 8.0, and 40% (3623 U g^−1^) at pH 5.0. No measurable activity was detected at pH 4.0 or 9.0. This narrow optimal range and sensitivity to pH highlight the importance of immobilization strategies to improve stability and operational flexibility.

Among the immobilized systems, ORG-PA exhibited the highest absolute activity, reaching 100% (287.7 U g^−1^) at pH 7.0—identical to the optimal pH of the free enzyme. This retention of the pH optimum suggests that adsorption onto the organic support did not significantly alter the enzyme’s protonation environment [12]. A similar result was reported by Almeida et al. [4], who immobilized *BCL* on guava seed biochar via physical adsorption, obtaining a hydrolytic activity of 260 U g^−1^ at pH 7.0. In the present study, ORG-PA maintained 57% (163.1 U g^−1^) and 75% (214.7 U g^−1^) of its maximum activity at pH 6.0 and 8.0, respectively. However, activity decreased sharply to 23% (66.9 U g^−1^) at pH 5.0 and was completely lost at pH 4.0 and 9.0. Despite its good performance at neutral pH, the system exhibited limited stability under more acidic or alkaline conditions.

The ORG-CB biocatalyst showed its highest activity at pH 8.0, 100% (213.8 U g^−1^), while still retaining 86% (183.8 U g^−1^) at pH 7.0 and 58% (124.0 U g^−1^) at pH 6.0. This slight shift in optimal pH toward more alkaline conditions, relative to the free enzyme, may be attributed to microenvironmental changes induced by covalent binding to the organic support. These effects, often associated with support polarity and surface charge, can influence the ionization of catalytic residues [4,12].

The HYB-PA system exhibited its maximum activity at pH 6.0, 100% (241.0 U g^−1^), followed by 66% (159.0 U g^−1^) at pH 5.0, 65% (156.5 U g^−1^) at pH 7.0, and 64% (155.2 U g^−1^) at pH 8.0. This profile reflects a broader functional pH range compared to ORG-PA, with good activity maintained from pH 5.0 to 8.0. The broader pH tolerance may be beneficial in processes subject to pH fluctuations. The shift in peak activity from neutral to slightly acidic conditions suggests that the hybrid support altered the enzyme’s microenvironment, likely due to its composite nature and heterogeneous surface chemistry, which may affect active site ionization.

In contrast, HYB-CB, INO-PA, and INO-CB demonstrated lower overall hydrolytic activity, despite showing moderate pH tolerance. Their lower absolute performance suggests possible limitations related to enzyme orientation, loading, or diffusional effects.

#### 2.1.3. Effect of Temperature on Hydrolytic Activity

**Figure 3:**
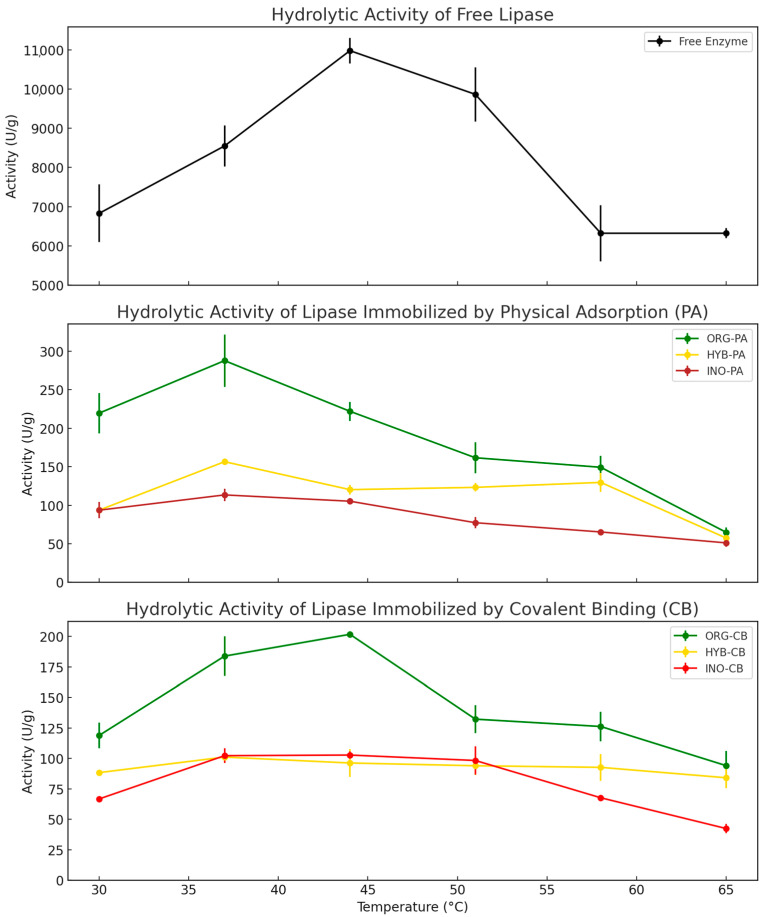
Effect of temperature on the Activity of Free and Immobilized *Burkholderia cepacia* Lipase (*BCL*) on organic (ORG), hybrid (HYB), and inorganic (INO) supports via physical adsorption (PA) and covalent binding (CB).

The effect of temperature on the hydrolytic activity of *BCL* was evaluated from 30 °C to 65 °C for both free and immobilized forms (Figure 3). The free enzyme exhibited maximum activity at 44 °C (10,984 U g^−1^), defined as 100% relative activity. High activity was also observed at 37 °C (8550 U g^−1^; 78%) and 51 °C (9868 U g^−1^; 90%), with slightly lower but still considerable values at 58 °C and 65 °C (both 6320 U g^−1^; 58%). This profile reflects the typical behavior of soluble lipases, which operate optimally in a moderately narrow range while maintaining substantial activity even at elevated temperatures.

Among the immobilized systems, the covalently bound derivatives demonstrated greater thermal resilience compared to physically adsorbed counterparts. HYB-CB exhibited the broadest thermal stability profile, retaining over 90% of its maximum activity up to 58 °C and 83% at 65 °C—surpassing the free enzyme, which retained only 58% at those temperatures. ORG-CB also showed improved stabilization relative to ORG-PA, maintaining 66% at 51 °C, 63% at 58 °C, and 47% at 65 °C. These findings support the role of covalent attachment in enhancing enzyme stability by restricting conformational flexibility and reducing thermal unfolding [4].

The inorganic systems (INO-PA and INO-CB) displayed the lowest overall performance. Although moderate retention was observed between 51 and 58 °C (57–68%), their peak activities (113.4 U g^−1^ and 102.3 U g^−1^, respectively) remained substantially lower than those of organic and hybrid systems—likely due to weak enzyme–support interactions and reduced substrate accessibility on silica matrices.

#### 2.1.4. Catalytic Performance

Beyond activity profiles, key kinetic and operational parameters were analyzed to compare immobilization efficiency. The kinetic parameters of free and immobilized *BCL* are summarized in Table 1. This evaluation is essential for understanding how immobilization affects catalytic performance. The main constants used in this analysis are the maximum reaction rate (Vmax), the Michaelis constant (Km), and the catalytic constant (Kcat). Vmax reflects the maximum rate at which an enzyme can convert substrate to product when all active sites are fully saturated, while Km represents the substrate concentration required to reach half of Vmax, serving as an indicator of the enzyme’s affinity for the substrate. Kcat corresponds to the turnover number, expressing how many substrate molecules are converted to product per minute by one enzyme molecule under saturation, and is calculated by dividing Vmax by the total amount of enzyme offered in the reaction system [10,13].

Changes in these parameters are expected following immobilization, as enzyme–support interactions may affect the orientation, flexibility, and accessibility of the active site [10]. Moreover, diffusion limitations—especially in porous or hybrid matrices—can restrict substrate access and raise Km while also contributing to reductions in Vmax. To better assess the impact of these effects, we also calculated the diffusion constant (Kα = Vmax/Km), which serves as an indicator of how efficiently the substrate can diffuse to and interact with the enzyme. Lower Kα values typically reflect mass transfer limitations or internal diffusion barriers, while higher values suggest more favorable substrate accessibility.

In addition, we evaluated the catalytic efficiency (Ke = Kcat/Km), which integrates the enzyme’s turnover capacity and substrate affinity. This parameter is especially relevant under non-saturating substrate conditions and reflects the enzyme’s real-world catalytic potential. Although Ke is widely used as a measure of catalytic efficiency, its interpretation must be context-specific. Eisenthal et al. [14] caution that comparing this parameter across different enzyme systems can be misleading unless kinetic conditions are tightly controlled. In the present study, since all immobilized systems derive from the same enzyme and were assayed under identical conditions, the use of Kcat/Km remains appropriate for assessing the relative impact of support composition on catalytic performance. To facilitate comparisons across systems, Ke values were also expressed relative to the free enzyme (Ke relative), highlighting how much catalytic performance was retained after immobilization.

As expected, the free enzyme exhibited the highest overall catalytic performance, with superior values across all kinetic parameters, reflecting full conformational freedom and unrestricted substrate access. Among the immobilized systems, those prepared by covalent binding consistently outperformed physically adsorbed counterparts across all support types, particularly in terms of lower Km, and higher relative catalytic efficiency (Ke) (Table 1). These values suggest that covalent attachment provided both structural stabilization and improved substrate interaction. This observation aligns with previous reports [10], in which covalent immobilization enhanced enzyme orientation, minimized leaching, and preserved catalytic conformation.

The best overall performance was observed for ORG-CB, which showed the lowest Km and the highest catalytic efficiency among all derivatives (Ke = 113.9 mM^−1^·min^−1^; 18.6% of the free enzyme). This indicates a favorable combination of substrate affinity and turnover rate, making it one of the most kinetically favorable immobilized systems tested. In contrast, although ORG-PA displayed the highest Vmax and Kcat, its extremely high Km (72,848.1 mM) led to poor relative catalytic efficiency (Ke = 0.010 mM^−1^·min^−1^; 2.9%), likely due to random enzyme orientation and limited substrate access resulting from the less controlled adsorption process.

Hybrid and inorganic systems exhibited lower catalytic efficiency overall, although covalently immobilized forms (HYB-CB and INO-CB) consistently outperformed their adsorbed counterparts in terms of Ke and relative Ke. Among these, INO-CB achieved a Ke of 98.9 mM^−1^·min^−1^ (16.2% relative), standing out as the second most catalytically efficient system after ORG-CB, though with a slightly higher Km and lower operational yield.

Taken together, these results indicate that ORG-CB offered the most balanced and effective catalytic behavior among all tested systems, justifying its selection for subsequent application in free fatty acid production from cocoa bean shell oil. It is important to note that kinetic parameters were determined using olive oil as a model substrate. Although CBS and olive oils present different fatty acid compositions, particularly in their content of saturated and unsaturated fatty acids—which can influence substrate binding and catalytic efficiency—olive oil, as a hydrolysis substrate, is widely used to compare the kinetic parameters of immobilized lipases. These results, together with other biochemical and physicochemical characterization data, are commonly used to select biocatalysts for future applications.

#### 2.1.5. Operational Stability (Reuse Cycles)

**Figure 4:**
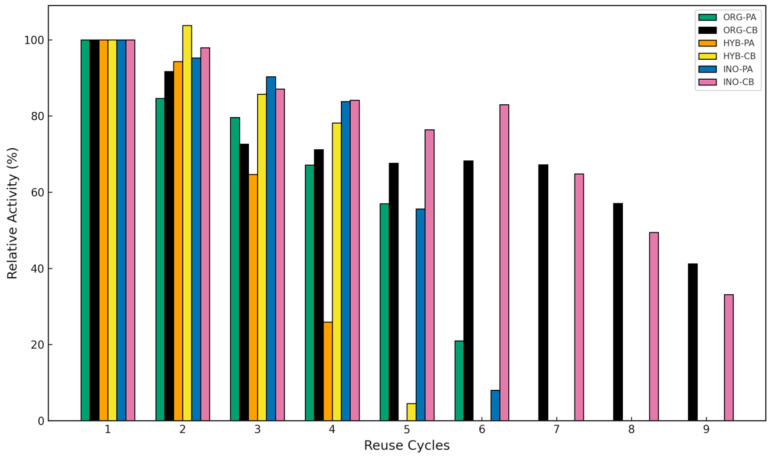
Operational stability of lipase immobilized by PA and CB on ORG, HYB, and INO supports.

The operational stability of *BCL* immobilized on organic, hybrid, and inorganic supports via physical adsorption (PA) and covalent binding (CB) was evaluated by monitoring its relative activity over consecutive reuse cycles (Figure 4). In all support categories, systems immobilized via covalent binding (CB) retained enzymatic activity for a greater number of reuse cycles compared to those immobilized by physical adsorption (PA).

For instance, the ORG-CB system maintained over 50% of its initial activity for eight cycles, whereas ORG-PA retained this level of activity for only five cycles. A similar trend was observed for hybrid and inorganic supports: HYB-CB vs. HYB-PA (four vs. three cycles) and INO-CB vs. INO-PA (seven vs. five cycles), respectively. This enhanced stability in CB systems can be attributed to the formation of strong covalent bonds between the enzyme and the support matrix, which minimizes enzyme leaching during repeated use [15]. In contrast, PA systems rely on weaker interactions—such as Van der Waals forces and hydrogen bonding—that are more susceptible to disruption, especially under agitation and prolonged use. These findings are consistent with literature reports, where covalent immobilization is often associated with greater resistance to desorption and higher operational durability of the biocatalyst [16,17].

Among all tested systems, ORG-CB exhibited the highest operational stability, making it the most promising strategy for applications requiring multiple enzyme reuse cycles. Its superior performance is likely due to both the chemical nature of the organic matrix and the robustness of the covalent immobilization process, which collectively contribute to the preservation of enzymatic activity over time.

#### 2.1.6. Integrated Evaluation and Selection of the Optimal Biocatalyst

Considering all evaluated parameters—activity under optimal pH and temperature, kinetic behavior, enzyme recovery, and reusability—the organic support with covalent binding (ORG-CB) was selected as the most promising immobilization system. It exhibited the highest immobilization yield (14.1%), indicating efficient enzyme anchoring and minimal structural damage during covalent attachment. This translated into favorable kinetic behavior, with the lowest Km among immobilized systems, suggesting enhanced substrate affinity and the highest relative catalytic efficiency (18.6%), reflecting effective catalysis under operational conditions. Notably, ORG-CB also demonstrated broad pH tolerance and strong thermal retention within the immobilized group, maintaining over 60% of its activity at 58 °C and 47% at 65 °C. While this performance did not surpass the free enzyme, it represents a meaningful stabilization relative to physically adsorbed forms. Additionally, ORG-CB displayed superior reusability, retaining over 50% of its activity for eight consecutive cycles. These findings indicate not only robust enzyme–support interaction but also preservation of conformational integrity, which is critical for process reliability. The alignment between high reuse stability and strong kinetic parameters reinforces the notion that effective immobilization enhances both enzyme retention and catalytic potential. In contrast, other systems showed trade-offs between stability, activity, and efficiency, with none matching the overall balance achieved by ORG-CB.

These integrated results confirm ORG-CB as the most promising candidate for biocatalytic applications involving the hydrolysis of cocoa bean shell oil, where performance, durability, and efficiency are equally essential.

### 2.2. Evaluation of Selected Biocatalyst

#### 2.2.1. Thermal Stability of Selected Biocatalyst

Kinetic and thermodynamic analyses provide information about the rate of enzymatic inactivation and parameters associated with catalytic efficiency and thermal stability. These characteristics are essential for designing cost-effective and time-efficient biocatalytic processes in industrial applications. The linearity of the Arrhenius plots, with coefficients of determination (R^2^) greater than 0.94, confirmed that thermal inactivation of both ORG-CB and free *BCL* followed first-order kinetics.

The thermal inactivation data for *BCL* reveal clear differences between the free enzyme and its covalently immobilized form on an organic support (ORG-CB) in terms of stability (Table 2). Kinetic parameters show that the ORG-CB had a slightly slower deactivation rate (kd = 0.0909 h^1^) and longer half-life (t_1/2_ = 7.63 h) than the free BLC (kd = 0.1001 h^−1^, t_1/2_ = 6.92 h) at 40 °C, suggesting some stabilization under mild thermal conditions. However, this trend reversed at higher temperatures: At 60 °C, the free enzyme outperformed the immobilized system, showing a half-life of 4.17 h compared to 3.62 h for ORG-CB. Both systems exhibited the expected thermal decay pattern, with increasing kd and decreasing t_1_/_2_ values as temperature rose. Notably, ORG-CB’s half-life decreased by over 50% between 40 and 60 °C, while the reduction for the free enzyme was less pronounced. These results indicate that although immobilization conferred some stability at lower temperatures, it did not significantly enhance short-term thermal stability in the 40–60 °C range.

In contrast, thermodynamic parameters of deactivation indicate that the immobilized enzyme is intrinsically more resistant to thermal denaturation. The deactivation enthalpy (ΔH‡) for ORG-CB remained constant around ~33.1–33.3 kJ mol^−1^ across all tested temperatures, while the free enzyme showed lower values (~28.0–28.1 kJ mol^−1^). More notably, the activation energy for thermal deactivation (Ed) increased from 21.95 kJ mol^−1^ for the free enzyme to 32.27 kJ mol^−1^ for the immobilized form, indicating a significantly greater energy barrier to unfolding or irreversible inactivation. A similar observation was reported by Jacob et al. [18], who obtained an Ed of 32.32 kJ mol^−1^ for *Candida rugosa* lipase immobilized on a SiO_2_/Fe_3_O_4_/GO nanocomposite. The increase in the positive enthalpic term of ORG-CB means that more energy is needed to denature the lipase protein. Those values are consistent with the structural constraints introduced by covalent attachment, which likely reduce conformational flexibility and enhance thermal rigidity [19].

The Gibbs free energy of deactivation (ΔG‡) was calculated and showed a similar pattern and is widely used as a complementary and reliable metric for evaluating enzyme stability [19,20]. ΔG‡ was consistently higher in the immobilized system, ranging from 6.24 to 4.5 kJ mol^−1^ from 40 to 60 °C, compared to 5.79 to 4.51 kJ mol^−1^ for the free enzyme. This indicates a less favorable inactivation pathway for the ORG-CB system. Furthermore, the entropy of deactivation (ΔS‡) was slightly more positive in the free enzyme (75–76 J mol^−1^·K^−1^) than in the immobilized system (approximately 74.3–74.8 J mol^−1^·K^−1^). These small differences suggest that while both systems undergo increased disorder in the transition state, the free enzyme experiences slightly greater conformational freedom during inactivation.

#### 2.2.2. Application: Hydrolysis of Cocoa Bean Shell Oil

Reconciling these findings, the effect of immobilization on thermal behavior is multifaceted. While ORG-CB displayed higher thermodynamic stability (higher Ed and ΔH‡), its kinetic stability—particularly under prolonged exposure to elevated temperatures—was not superior to the free enzyme. This suggests that immobilization increased the energy barrier for thermal inactivation but also made the inactivation process more sensitive to temperature. For industrial applications, such trade-offs underscore the need to carefully align immobilization strategies with process temperature demands, especially when balancing enzyme reuse with sustained activity over time.

The fatty acid composition of cocoa bean shell oil was determined by gas chromatography with flame ionization detection (GC-FID) following methyl ester derivatization. Table 3 presents the identified fatty acids, along with their molar masses, mass percentages, and corresponding mole percentages (mmol%). The oil showed a balanced profile of saturated and unsaturated fatty acids. Palmitic acid (C16:0) and stearic acid (C18:0) were the predominant saturated species (28.0% and 23.2%, respectively), while oleic acid (C18:1) stood out among the unsaturated ones (35.7%), followed by linoleic (11.8%) and linolenic acid (0.7%). These results are in agreement with literature reports that highlight the abundance of oleic, palmitic, and stearic acids in cocoa-derived lipids [21]. The compositional data also enabled the calculation of the average molar mass of the oil, estimated at 275.59 g/mol based on weighted fatty acid contributions. This value is fundamental for converting oil mass into theoretical moles of fatty acids released upon complete hydrolysis, providing a stoichiometric basis for evaluating enzymatic conversion.

The enzymatic hydrolysis of cocoa bean shell oil by *BCL* exhibited a markedly higher efficacy when the enzyme was immobilized on an organic support via covalent bound (ORG-CB) compared to its free form. In our experiments, the free enzyme yielded only about 49.9% conversion of the oil to free fatty acids, whereas the immobilized enzyme achieved a conversion of 60.1%. This improvement is consistent with some reports in the literature where immobilized lipases often outperform their free counterparts in lipid transformations [22,23]. The higher conversion attained with the immobilized lipase can be attributed to several factors, including enhanced stability of the enzyme under reaction conditions, improved enzyme-substrate contact at the oil–water interface, and reduction in diffusional limitations or inhibitory effects. In contrast, the free enzyme likely suffered from limited interaction with the hydrophobic substrate and/or partial inactivation during the reaction, leading to a relatively low extent of hydrolysis.

Willerding et al. [24] employed a Central Composite Design (CCD) to optimize the enzymatic hydrolysis of several Amazonian vegetable oils using bacterial lipases. The hydrolysis of oils by *BCL* was evaluated under varying conditions of temperature (31.6–48.4 °C), enzyme concentration (1–5%), and oil-to-buffer ratio (1:1 to 1:5), aiming to maximize the production of free fatty acids (FFAs). The FFA production from buriti oil ranged from 9% to 16%, while babassu oil presented much higher yields (40–88%). In a recent study, Santos et al. [25] investigated the enzymatic hydrolysis of babassu oil using combinations of immobilized lipases: *Rhizomucor miehei* (RML-IM), *Candida antarctica B* (Novozym^®^ 435), and *Thermomyces lanuginosus* (TLL-IM). They reported a 58% hydrolysis yield after 1 h using 100% RML-IM. Remarkably, a combination of 50% RML-IM and 50% Novozym 435 achieved over 99% hydrolysis after 4 h. These results highlight the effectiveness of immobilized lipases and their combinations in achieving high FFA conversions, underscoring the potential of tailored biocatalyst formulations in enhancing hydrolysis efficiency.

### 2.3. Morphological and Physicochemical Characterization of Selected Biocatalyst

#### 2.3.1. FTIR Analysis

**Figure 5:**
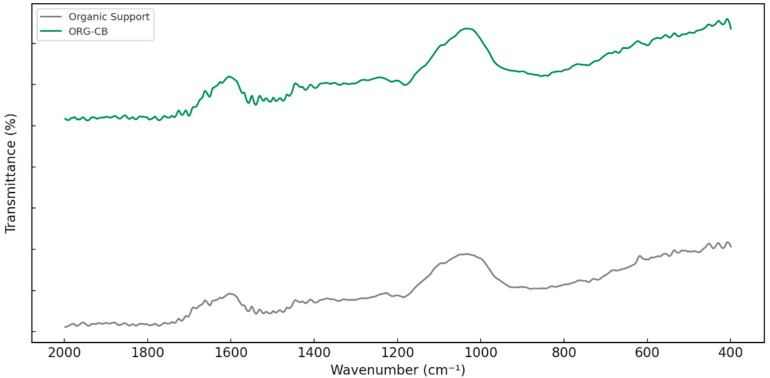
FTIR spectra for ORG-CB and silanized organic support.

FTIR spectra were recorded in transmittance mode within the range of 400 to 2000 cm^−1^, which encompasses the key functional group vibrations relevant to this study (Figure 5). The spectra of the silanized organic support and the immobilized biocatalyst (ORG-CB) revealed spectral features consistent with the presence of APTES and the successful immobilization of lipase. Bands around 1450 and 1390 cm^−1^ present in both spectra correspond to the bending vibrations of methylene and methyl groups introduced by the propyl chains of APTES. These assignments align with observations reported by Jacob et al. [18], who attributed similar bands to covalently bound APTES on silica-based composites.

Spectral differences between the support and ORG-CB were evident in the amide region. A shoulder near 1650 cm^−1^, present only in the ORG-CB spectrum, was attributed to the C=O stretching vibration of Amide I. Furthermore, the band near 1510 cm^−1^ showed increased intensity in ORG-CB, suggesting the presence of N–H bending (Amide II). In addition, the ORG-CB spectrum retains the aliphatic C–H bending bands (~1460 and ~1390 cm^−1^), with even slightly increased intensity relative to S2. This persistence and enhancement of the –CH_2_/–CH_3_ signals further indicate the addition of organic (protein) matter on the surface. Together, the appearance of strong amide absorptions and the increase in aliphatic C–H bands in the ORG-CB sample confirm that the enzyme has been successfully immobilized onto the silanized support. The appearance of amide bands in the ORG-CB spectrum provides strong evidence for successful covalent immobilization. These spectral changes, particularly the emergence of Amide I and II signals, are consistent with enzyme attachment and have been widely reported as reliable markers of covalent binding [4,18,26].

#### 2.3.2. Morphology Study of Support and ORG-CB Biocatalyst by SEM

**Figure 6:**
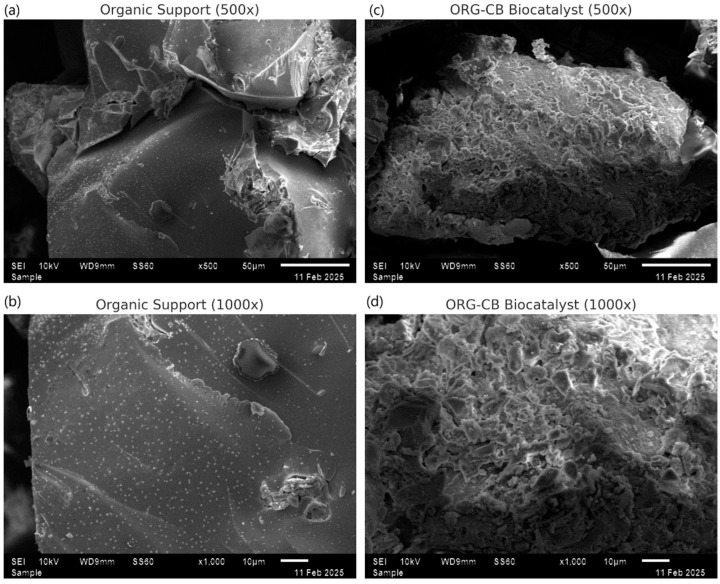
SEM of silanized organic support (**a**,**b**) and ORG-CB biocatalyst (**c**,**d**) at 500× and 1000×, respectively.

Scanning electron microscopy (SEM) was employed to investigate the morphological differences between the silanized organic support and the ORG-CB biocatalyst at two magnifications (500× and 1000×) (Figure 6). At 500×, the organic support exhibited a relatively uniform surface, with visible fibrous structures typical of lignocellulosic materials. In contrast, the ORG-CB biocatalyst displayed a denser and more compact surface with irregular coverage, suggesting the formation of a superficial layer resulting from the covalent attachment of *BCL*.

At higher magnification (1000×), the differences between the two materials became even more evident. The support maintained its smooth and open texture, while the biocatalyst exhibited increased roughness, heterogeneity, and the presence of granular deposits. These features are consistent with the adsorption and crosslinking of enzyme molecules over the support matrix. The changes in surface topography observed at both magnifications reinforce the conclusion that immobilization led to significant alterations in the material’s structure, providing qualitative evidence of successful enzyme anchoring. Such morphological changes are commonly reported in the literature and are often associated with enhanced biocatalyst stability and performance [4].

#### 2.3.3. Thermogravimetric Analysis (TGA) and Derivative Thermogravimetry (DTG)

TGA is a technique usually employed for the analysis of the decomposition and thermal stability of materials. In this study, TGA was used to assess the thermal stability and confirm the presence of *BCL* immobilized on a silanized organic support via covalent bonding. Comparative analyses were performed on the bare support and the resulting biocatalyst (ORG-CB). The corresponding DTG curves were also examined to better resolve degradation events.

**Figure 7:**
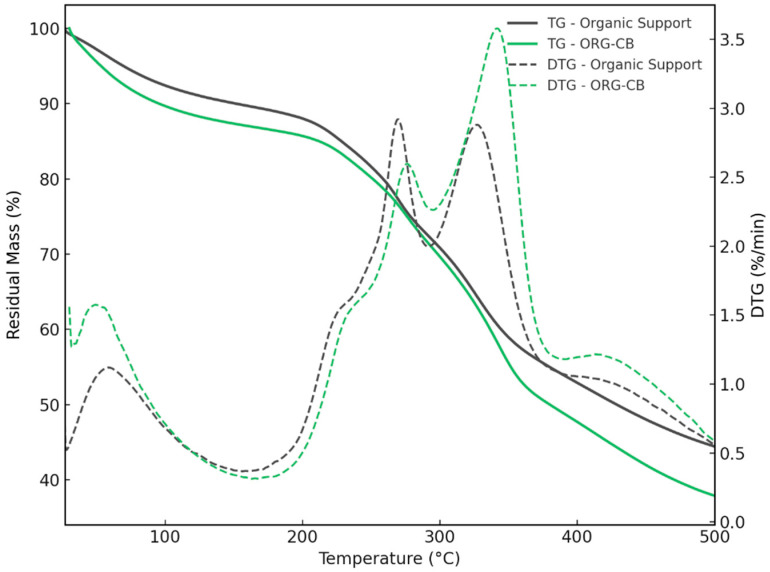
Thermogravimetric (TGA) and derivative thermogravimetric (DTG) analysis of silanized organic support and ORG-CB biocatalyst.

The curves in Figure 7 show that ORG-CB and the silanized support are degraded in a three-stage process. The first stage occurred between 35 and 100 °C, resulting from the removal of water, corresponding to a mass loss of less than 5% for both materials.

A more pronounced mass loss occurred between 200 and 400 °C, associated with the decomposition of the organic matrix and, in the case of ORG-CB, the immobilized enzyme. In this range, the ORG-CB system exhibited a mass loss of 14.3%, compared to 12.0% for the bare support—corresponding to an additional 2.3% degradation attributed to the enzyme. This distinction is further supported by the DTG curves (Figure 7), where ORG-CB presents a more intense degradation peak centered around 305 °C, consistent with the thermal breakdown of protein structures [27,28,29]. The final stage occurs above 400 °C and involves the decomposition of more thermally stable components and the formation of residual char.

These results confirm the successful immobilization of the enzyme and demonstrate that the biocatalyst retains structural stability up to approximately 200 °C. It is important to emphasize, however, that this thermal resistance does not imply preservation of catalytic activity at elevated temperatures. Indeed, as shown in hydrolytic activity assays, both the free and immobilized enzymes displayed optimal activity between 37 °C and 44 °C, with a marked decline above 58 °C. Nonetheless, the high decomposition temperature observed in TGA suggests that the enzyme is stably anchored to the support, and the overall structure of the biocatalyst is robust—an important feature for handling, storage, and certain industrial applications where temperature fluctuations may occur outside the enzyme’s optimal catalytic range.

#### 2.3.4. X-Ray Diffraction (XRD)

**Figure 8:**
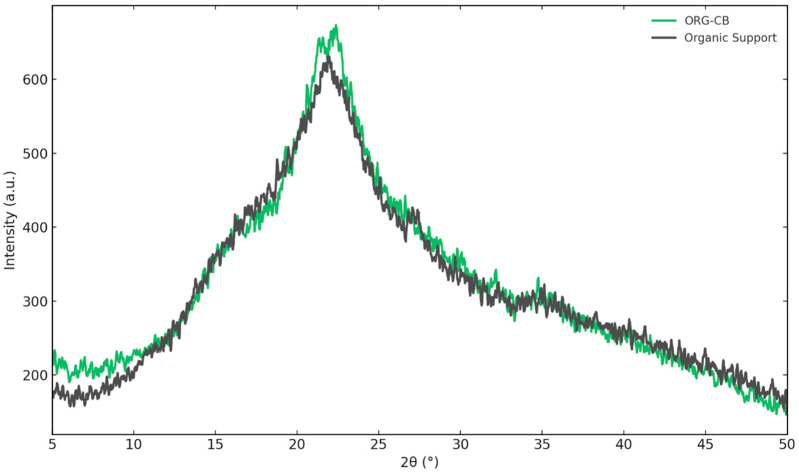
XRD patterns of silanized organic support and ORG-CB biocatalyst.

XRD patterns of both the silanized organic support and the ORG-CB biocatalyst exhibit broad, diffuse halos rather than sharp diffraction peaks, indicative of an amorphous structure (Figure 8). This behavior is typical of lignocellulosic materials and suggests a disordered molecular arrangement favorable for enzyme immobilization. The retention of the amorphous profile after immobilization indicates that the incorporation of the enzyme did not alter the structural morphology of the support, maintaining high surface accessibility and flexibility—both beneficial traits for catalytic activity and diffusion.

## 3. Materials and Methods

### 3.1. Materials

Cocoa bean shells were donated by a chocolate factory located in Salvador, Bahia, Brazil, according to the geographic coordinates: latitude −12°54′13.6728″, −38°26′34.3926″.

Amano Lipase from *Burkholderia cepacia* (*BCL*) was purchased from Sigma Chemical Co. (St. Louis, MO, USA). Nominal lipase activity was >30,000 U g^−1^. Hexane and acetone were obtained from Isofar (Rio de Janeiro, Brazil); 95% ethanol was obtained from Vetec (Rio de Janeiro, Brazil); Arabic gum was obtained from Cromoline (São Paulo, Brazil); virgin olive oil was purchased at a local market (Salvador, Brazil). Other chemicals were of analytical grade and used as received.

### 3.2. Cocoa Bean Shell Oil Extraction and Characterization

#### 3.2.1. Oil Extraction from Cocoa Bean Shells

Prior to lipid extraction, the water content of the cocoa bean shell samples was reduced by drying at approximately 60 ± 2 °C until the moisture content was below 10%. For oil extraction, 20 g of dried cocoa bean shells were subjected to Soxhlet extraction using 150 mL of hexane under reflux for 8 h. Following extraction, the solvent was removed by rotary evaporation at 60 °C, and the crude oil was subsequently recovered.

#### 3.2.2. Fatty Acid Composition of Cocoa Bean Shells

The fatty acid profile was determined by a capillary column gas chromatographic method according to Joseph & Ackman [30]. The separation of the methyl esters in the fatty acids was performed using gas chromatography (Varian 3800, Palo Alto, CA, USA) with a flame ionization detector (GC-FID) and a fused silica gas chromatography capillary column Elite WAX (30 m × 0.32 mm × 0.25 μm) (PerkinElmer, Norwalk, CT, USA), split injection (1:100), injector temperature at 250 °C, detector temperature at 280 °C, column temperature maintained at 150 °C for 16 min, and programmed to increase 2 °C per min until 180 °C, remaining at this temperature for 25 min and then programmed to increase 5 °C per min until 210 °C, remaining at this last temperature for 25 min.

The quantification of fatty acids, expressed in milligrams per 100 g sample, was executed by the addition of an internal standard (C23:0 Sigma, St. Louis, MO, USA) according to Joseph & Ackman [30] and calculated using Equation (1).

(1) $$Concentration\left(mg/100\;g\;of\;sample\right)\;=\frac{A_{FA}\;*\;M_{IS}\;*\;F\;*\;C_{TL}}{A_{IS}\;*\;M\;*\;FFA}$$

where *A_FA_* = area of fatty acid methyl ester peak in the chromatogram of the sample; *M_IS_* = weight (in milligrams) of the internal standard added to the sample; *F* = correction factor of fatty acid methyl ester to fatty acid; *C_TL_* = percentage composition of total lipids from the sample; *A_IS_* = area of internal standard fatty acid methyl ester peak in the chromatogram of the sample; M = sample mass (in milligrams); *F_FA_* = correction factor response of each fatty acid methyl ester ionization detector, relative to C23:0.

### 3.3. Support Preparation

#### 3.3.1. Organic Support

The organic support used in this study consisted of defatted cocoa bean shells, which were previously dried and sieved to obtain particle sizes ranging from 20 to 30 mesh. No additional chemical treatment or surface activation was applied to the CBS used in immobilization by physical adsorption, preserving the native structure of the lignocellulosic matrix. In contrast, for covalent binding, the CBS support was subjected to surface functionalization via silanization (Section 3.4.2). Specifically, CBS was treated with 3-aminopropyltriethoxysilane (APTES) to introduce amine groups onto the support surface, followed by activation with glutaraldehyde.

#### 3.3.2. Inorganic Support

The inorganic support was synthesized using the sol–gel method, following the procedure described by Mota et al. [31], with modifications. Briefly, 30 mL of tetraethoxysilane (TEOS) was diluted in 36 mL of absolute ethanol (99%) under a nitrogen atmosphere and stirred at room temperature for approximately 5 min. Then, 0.22 mL of hydrochloric acid solution (36%, *v/v* in ultra-pure water) was slowly added to initiate pre-hydrolysis. The mixture was stirred at 200 rpm for 90 min at 35 °C. Afterward, the hydrolysis was completed by adding 1 mL of ammonium hydroxide dissolved in 6 mL of ethanol. The solution was kept under static conditions for 24 h to allow gelation. The resulting gel was then removed, washed with hexane, and stored in a desiccator for 72 h.

#### 3.3.3. Hybrid Support

The hybrid support was prepared using the same sol–gel method as described for the inorganic support [31], with the inclusion of cocoa bean shell powder. Specifically, 3.0 g of defatted and sieved cocoa bean shells were added to the reaction mixture before the hydrolysis step. The process then continued as described for the inorganic support, including the addition of ammonium hydroxide, static gelation, washing with hexane, and drying in a desiccator for 72 h.

### 3.4. Lipase Immobilization

Amano lipase from *Burkholderia cepacia* was immobilized by two different techniques —physical adsorption and covalent binding—on three types of supports: an organic support (cocoa bean shell), an inorganic support (silica), and a hybrid support composed of silica and cocoa bean shell. This resulted in six immobilized enzyme systems: lipase physically adsorbed on the organic (ORG-PA), hybrid (HYB-PA), and inorganic (INO-PA) supports, and lipase covalently bound to the organic (ORG-CB), hybrid (HYB-CB), and inorganic (INO-CB) supports.

#### 3.4.1. Immobilization by Physical Adsorption (PA)

Immobilization by physical adsorption was carried out following the method described by Soares et al. [21], with slight modifications. Briefly, 1.0 g of the dried support was suspended in 15 mL of hexane and subjected to mechanical stirring for 15 min. Then, 10 mL of an aqueous solution containing 0.3 g of free lipase (0.03 g/mL) was added to the suspension, resulting in a final enzyme-to-support ratio of 0.3 g enzyme/g support. The mixture was stirred for 3 h at room temperature. After this period, the immobilized enzyme preparation was stored at 4 °C for 24 h. Subsequently, the biocatalyst was washed with distilled water, vacuum filtered, and dried in a desiccator for 24 h.

#### 3.4.2. Immobilization by Covalent Binding (CB)

Prior to immobilization, the supports were silanized by refluxing 1.0 g of dried support in a 0.5% (*v/v*) solution of 3-aminopropyltriethoxysilane (APTES) at 75 °C for 2 h. Following silanization, the supports were activated with 4.6 mL of a 2.5% (*v/v*) glutaraldehyde solution in distilled water for 1 h at room temperature. The activated supports were then washed with three 10 mL portions of distilled water, vacuum filtered, and dried in a desiccator for 12 h. After silanization and activation, the lipase was immobilized using the same procedure described at Section 3.4.1.

### 3.5. Biochemical Characterization of Free and Immobilized Lipase Systems

Olive oil was used as a model substrate for the enzymatic characterization and comparative evaluation of the immobilized systems due to its well-established profile and suitability for activity assays [4,21,31,32]. Following the selection of the most efficient biocatalyst based on these parameters, cocoa bean shell oil—a waste-derived and chemically distinct substrate—was employed in the final hydrolysis application to evaluate the potential of the system for sustainable free fatty acid (FFA) production.

#### 3.5.1. Determination of Hydrolytic Activity and Immobilization Yield

The hydrolytic activity of free and immobilized lipase was determined using the olive oil emulsion method, according to the procedure described by Soares et al. [21], with some modifications. The reaction mixture consisted of 1 mL of olive oil emulsion (prepared by mixing equal volumes of olive oil and 7% (*w/v*) Arabic gum solution), 1 mL of sodium phosphate buffer (0.1 M, pH 7.0), and 0.3 mL of either lipase solution (for the free enzyme system) or biocatalyst suspension (for the immobilized systems). Reactions were carried out in a water bath at 37 °C under constant agitation, with incubation times of 5 min for the free enzyme and 10 min for the immobilized systems. The reaction was stopped by the addition of 3 mL of an acetone–ethanol (1:1, *v/v*) mixture, and the amount of free fatty acids released was titrated with 0.02 M KOH using phenolphthalein as an indicator. One unit (U) of enzyme activity was defined as the amount of enzyme required to release 1 µmol of fatty acids per minute under the assay conditions. Analyses of hydrolytic activities performed on free and immobilized lipase were used to determine the immobilization yield (%) according to Equation (2) [10].

(2) $$RI\;\left(\%\right)=\frac{Us}{Uo}\;*\;100$$

in which *Us* corresponds to the total enzyme activity recovered on the support, and *Uo* represents the enzyme units offered for immobilization.

#### 3.5.2. Effect of pH and Temperature on Activity

The effect of pH on the activity of free and immobilized lipase was determined in buffers with values between pH 4 and 9. The buffers used were 0.1 M citric acid–sodium citrate (pH 4.0–5.0), 0.1 M potassium phosphate (pH 6.0–8.0), and 0.1 M bicarbonate–carbonate (pH 9.0–10). The optimal temperature for the activity of free and immobilized lipase was assayed in the 30–65 °C range in the same 0.1 M potassium phosphate buffer (pH 7.0) [30].

#### 3.5.3. Determination of Kinetic Constants

The kinetic constants of Michaelis–Menten (Km and Vmax) were determined by different fatty acid concentrations between 125 and 775 mM in reaction systems. This was obtained from emulsions containing different proportions of olive oil (10–55% *v/v*) and aqueous solution of 7% (*w/v*) Arabic gum solution. The apparent values of Km and Vmax were calculated according to the linearization methods of Lineweaver–Burk [30]. Their derivative constants (catalytic constant–Kcat, diffusion constant–Kα, and specific constant–Ke) were determined according to Equations (3)–(5). [10,14].

Kcat = Vmax/[E]_0_ (3)

Kα = Vmax/Km(4)

Ke = Kcat/Km(5)

where [E]_0_ corresponds to the initial concentration of enzyme offered in the reaction.

#### 3.5.4. Operational Stability (Reuse Cycles)

The operational stability of the immobilized systems was assayed by running hydrolysis reactions in consecutive batches using the same biocatalyst. The time of each hydrolysis reaction was 10 min at a temperature of 37 °C and pH of 7.0.

### 3.6. Evaluation of Selected Biocatalyst

#### 3.6.1. Thermal Stability Assessment

The thermodynamic assessments of enzyme inactivation were adapted from Almeida et al. [4] and Elias et al. [19], with some modifications. The thermal stability of both free and immobilized lipase (ORG-CB) was evaluated by incubating the biocatalysts in sodium phosphate buffer (0.1 M, pH 7.0) at 40, 50, and 60 °C for 24 h. Aliquots were withdrawn at 1, 2, 4, 6, 8, 12, and 24 h to monitor residual enzymatic activity.

The thermal inactivation rate constant (*k_d_*, h^−1^) was determined based on first-order kinetics using Equations (6) and (7), where A represents the residual activity (U) at time t, and A0 is the initial activity (U):

(6) $$A=A0\exp\left(K_{d}\cdot t\right)$$

(7) $$\ln\left(\frac{A}{A0}\right)=-K_{d}\cdot t$$

Values of *k_d_* for both free *BCL* and ORG-CB at each temperature were calculated from the slope of the linear regression of ln(*A/A*0) versus time. The half-life (*t*_1/2_) of enzymatic activity, defined as the time required for the activity to decrease by 50%, was calculated using Equation (8):

(8) $$t_{1/2}=\frac{\ln\left(2\right)}{K_{d}}$$

The activation energy for thermal deactivation (Ed, kJ·mol^−1^) was obtained from the slope of the Arrhenius plot by plotting *ln*(*K_d_*) vs. 1/*T* according to Equation (9), where R is the universal gas constant (8.314 J·mol^−1^·K^−1^), and T is the absolute temperature (K):

(9) $$\ln\left(K_{d}\right)=\ln\left(A\right)-\frac{E_{d}}{R}\cdot \frac{1}{T}$$

The thermodynamic parameters relating to enthalpy of deactivation (ΔH‡), Gibb’s free energy (ΔG‡), and change in entropy (ΔS‡) were estimated using Equations (10)–(12), respectively [18,19]:

(10) $$\Delta H‡\;=E_{d}-RT$$

(11) $$\Delta G‡=-RT\ln\left(k_{d}\right)$$

(12) $$\Delta S‡=\frac{\left(\Delta H‡-\Delta G‡\right)}{T}$$

#### 3.6.2. Application: Hydrolysis of Cocoa Bean Shell Oil

After selecting the most efficient biocatalyst based on characterization with olive oil, cocoa bean shell oil was used as a real substrate for free fatty acid (FFA) production. The hydrolytic conversion of cocoa bean shell oil into FFAs by free and immobilized *BCL* was evaluated by titration, following the method described by Soares et al. [21], with some modifications. The reaction mixture consisted of 1 mL of cocoa bean shell oil emulsion (prepared by mixing equal volumes of cocoa bean shell oil and 7% (*w/v*) Arabic gum solution), 1 mL of sodium phosphate buffer (0.1 M, pH 7.0), and 0.3 mL of either lipase solution (for the free enzyme system) or biocatalyst suspension (for the immobilized systems). Reactions were carried out in a water bath at 37 °C for 7 h. After incubation, the reaction was stopped by the addition of 3 mL of an acetone:ethanol mixture (1:1, *v/v*).

The mixture was titrated with 0.1 M KOH using phenolphthalein as a pH indicator. A blank (without enzyme) was titrated under the same conditions to account for the initial FFA content of the oil. The *FFA* content was calculated from the volume of KOH consumed, and the percentage of conversion was estimated (Equation (13)):

(13) $$FFA\;Conversion\;\left(\%\right)=\;\frac{\left(Vsample-Vblank\right)\;*\;f\;*\;M}{mmolFFAtheoretical}$$

where *Vsample* = volume (mL) of KOH used in the sample, *Vblank* = volume (mL) of KOH used in the blank, *M* = molarity of KOH, *f* = correction factor from standardization against potassium biphthalate (dimensionless), *mmolFFAtheoretical* = theoretical amount of FFAs that would be produced assuming complete hydrolysis of the oil (based on the stoichiometry 1:3, i.e., 1 mmol of triglyceride yields 3 mmol of FFAs).

#### 3.6.3. Physicochemical and Morphological Characterization

Samples of free lipase, support, and immobilized derivative were submitted to FTIR analysis (IR Prestige 21, Shimadzu, Tokyo, Japan). FTIR spectra were recorded for the support and selected immobilized enzyme in the 4000–400 cm^−1^ range. However, in the fingerprint region (2000–400 cm^−1^), a more resolved secondary dataset was obtained and used for detailed band assignments. Morphology was analyzed using scanning electron microscopy (Hitachi S-3000N, Tokyo, Japan). Thermogravimetric analysis (TGA) was performed using 5.0 mg of the samples supported in an alumina crucible, using a flow rate of 10 mL min^−1^ and heating rate of 10 °C/min, from 35 °C to 500 °C under nitrogen atmosphere. Energy dispersive X-ray analysis (EDX) (Rayny EDX 700, Shimadzu, Tokyo Japan) was used as the support and immobilized derivative.

## 4. Conclusions

This study demonstrated the potential of cocoa bean shells (CBSs), an underutilized agro-industrial byproduct, to function simultaneously as an enzyme immobilization support and as a reaction substrate in a sustainable biocatalytic system. Among the six evaluated systems, the covalently immobilized lipase on CBS (ORG-CB) presented the best overall performance, combining favorable kinetic parameters, high reuse stability, and efficient fatty acid production from residual cocoa oil. Thermodynamic analysis revealed that covalent immobilization enhanced the structural rigidity of the enzyme, though it also introduced some limitations in thermal deactivation kinetics.

The successful conversion of cocoa bean shell oil into free fatty acids—achieving 60.1% hydrolysis—demonstrates the practical value of CBS as both a support and a renewable feedstock. These results validate a dual-purpose approach aligned with circular economy principles, enabling the valorization of food industry waste while advancing greener enzyme-based technologies. The findings also emphasize the critical importance of immobilization strategy and support selection in tuning biocatalyst performance for industrial applications.

## Figures and Tables

**Table 1:** Catalytic performance of free and immobilized *Burkholderia cepacia* lipase (*BCL*).

System	Vmax (µmol·min^−1^)	Km (mM)	Kcat (min^−1^)	Kα (min^−1^)	Ke (mM^−1^·min^−1^)	Relative Ke (%)
Free Enzyme	21.7	599.0	202.5	36.2	0.338	100.0
ORG-PA	752.1	72,848.1	702.0	10.3	0.010	2.9
ORG-CB	5.6	83.4	5.2	67.4	0.063	18.6
HYB-PA	35.9	5303.9	33.5	6.8	0.006	1.9
HYB-LC	2.3	287.4	2.2	8.1	0.008	2.2
INO-PA	8.1	1320.0	7.6	6.2	0.006	1.7
INO-LC	8.9	151.5	8.3	58.5	0.055	16.2

**Table 2:** Comparison of inactivation rate constants (kd), half-life (t_1/2_), and thermodynamic parameters for free and immobilized *Burkholderia cepacia* lipase (ORG-CB).

System	Temperature (°C)	kd (h^−1^)	t_1_/_2_ (h)	ΔH‡ (kJ mol^−1^)	ΔG‡ (kJ mol^−1^·K^−1^)	ΔS‡ (kJ mol^−1^)	Ed (kJ mol^−1^)
	40	0.091	7.63	33.30	6.24	74.80	
ORG-CB	50	0.125	5.54	33.22	5.58	74.27	35.90
	60	0.192	3.62	33.14	4.57	74.82	
	40	0.100	6.92	28.05	5.79	76.23	
Free lipase	50	0.125	5.53	27.96	5.22	75.38	60.65
	60	0.166	4.17	27.88	4.51	75.00	

**Table 3:** Fatty acid profile of cocoa bean shell oil.

Fatty Acid	Cx:y ^a^	MM (g.mol^−1^)	% mass ^b^	% mmol
Palmitic	C16:0	256.43	28 ± 1	30.2 ± 1.08
Stearic	C18:0	284.49	23.2 ± 0.8	22.6 ± 0.78
Oleic	C18:1	282.47	35.7 ± 1	35.0 ± 0.98
Linoleic	C18:2	282.47	11.8 ± 0.04	11.6 ± 0.04
Linolenic	C18:3	280.45	0.7 ± 0.1	0.7 ± 0.10
Average molar mass MM (g.mol^−1^)			275.59	
Saturated/Unsaturated Ratio (by mass)			1.06	

^a^ Cx:y, x = number of carbon atoms; y = number of double bonds; ^b^ fat extracted from cocoa bean shell oil using Soxhlet system.

## Data Availability

The original contributions presented in this study are included in this article; further inquiries can be directed to the corresponding author.

## Abbreviations

The following abbreviations are used in this manuscript:

TAG	Triacylglycerol
FFA	Free fatty acids
CBSs	Cocoa bean shells
*BCL*	*Burkholderia cepacia* Lipase
ORG	Organic support (Cocoa bean shell-based)
HYB	Hybrid support (Cocoa shells silica composite)
INO	Inorganic support (Silica)
PA	Physical adsorption
CB	Covalent binding
Vmax	Maximum reaction rate
Km	Michaelis–Menten constant
Ke	Catalytic efficiency
Kα	Diffusion constant
Kd	Thermal deactivation constant
Ed	Deactivation activation energy
ΔH	Enthalpy of activation for thermal deactivation
ΔG	Gibbs free energy of activation for thermal deactivation
ΔS	Entropy of activation for thermal deactivation
U	Enzyme activity units
SEM	Scanning electron microscopy
FTIR	Fourier-transform infrared spectroscopy
TGA	Thermogravimetric analysis
XRD	X-ray diffraction
